# Osteopontin/SPP1: a potential mediator between immune cells and vascular calcification

**DOI:** 10.3389/fimmu.2024.1395596

**Published:** 2024-06-11

**Authors:** Yanli Zhao, Zujuan Huang, Limei Gao, Hongbo Ma, Rong Chang

**Affiliations:** ^1^ Department of Cardiovascular Medicine, Shenzhen Longhua District Central Hospital, Shenzhen, China; ^2^ School of Clinical Medicine, Chengdu University of Traditional Chinese Medicine, Chengdu, Sichuan, China

**Keywords:** OPN, SPP1, immune cells, vascular calcification, vascular diseases

## Abstract

Vascular calcification (VC) is considered a common pathological process in various vascular diseases. Accumulating studies have confirmed that VC is involved in the inflammatory response in heart disease, and SPP1+ macrophages play an important role in this process. In VC, studies have focused on the physiological and pathological functions of macrophages, such as pro-inflammatory or anti-inflammatory cytokines and pro-fibrotic vesicles. Additionally, macrophages and activated lymphocytes highly express SPP1 in atherosclerotic plaques, which promote the formation of fatty streaks and plaque development, and SPP1 is also involved in the calcification process of atherosclerotic plaques that results in heart failure, but the crosstalk between SPP1-mediated immune cells and VC has not been adequately addressed. In this review, we summarize the regulatory effect of SPP1 on VC in T cells, macrophages, and dendritic cells in different organs’ VC, which could be a potential therapeutic target for VC.

## Introduction

Vascular diseases, particularly cardiovascular and brain diseases, are the leading causes of human disease mortality. Vascular calcification (VC) is considered a common pathological process in various vascular diseases, such as diabetes (1), atherosclerosis (2, 3), vascular injury (4, 5), chronic kidney disease (CKD) (6), liver fibrosis (7), and aging (8), and is closely associated with chronic calcium-phosphate deposition in blood vessels (9, 10). Intimal calcification and medial calcification are two types of VC; the former is closely related to the infiltration of inflammatory cells, vascular inflammation, lipid deposits, hyperlipidemia, and hypertension, and the latter is associated with aging, diabetes, CKD, and arterial stiffness (11, 12). The inflammatory response plays a vital role in different VCs. Pro-inflammatory macrophages contribute to microcalcification though intimal extracellular matrix (ECM) degradation and mineralization, which are closely related to the production of IL-1β, TNFα, and IL-18. Microcalcification induces an osteoblast-like phenotype in vascular smooth muscle cells (VSMCs) and then enhances the production of inflammatory cytokines, such as IL-1β, IL-6, and osteopontin (OPN), resulting in more dense calcification in atherosclerotic plaques (13).

SPP1 (Secreted Phosphoprotein 1 or OPN), a macrophage-derived OPN, plays an important regulatory role in cardiac repair after myocardial injury and pathological cardiac hypertrophy (14). After myocardial infarction, infiltrated macrophages are found in the myocardial infarction site, and the expression of SPP1, which is derived from macrophages, increases, but SPP1 is not expressed in normal cardiac tissue (15). The data in the SPP1 knockout mouse model revealed that loss of the SPP1 gene does not affect cardiac function but results in a decrease in collagen (16). In addition, the expression of the SPP1 gene is positively correlated with cardiac hypertrophy, which causes cardiomyocyte apoptosis and fibrosis (17). Studies in vascular endothelial cells have shown that SPP1 promotes angiogenesis and endothelial migration (18, 19). Excessive SPP1 is involved in the proliferation and migration of VSMCs, leading to vascular hyperplasia (20, 21). In addition, macrophages and activated lymphocytes highly express SPP1 in atherosclerotic plaques, which promote the formation of fatty streaks and plaque development, and SPP1 is also involved in the calcification process of atherosclerotic plaques and results in heart failure (22).

Single-cell transcriptomics analysis revealed that macrophages, natural killer T cells, and T and B lymphocytes are major immune cell subsets in calcified atheromatous plaques in asymptomatic patients (23). Additionally, an increasing number of studies have suggested that SPP1 participates in different inflammatory responses to VC by regulating immune cells. TREM2^hi^ (triggered receptor expressed on myeloid cells 2) macrophages display a unique gene signature with expression of SPP1 in mouse atherosclerotic lesions (24). Compared with those in the normal group, SPP1 is positively correlated with dendritic cells (DCs) and regulatory T cells in the calcific aortic valve disease (CAVD), but there is a negative correlation between SPP1 and M2 phenotype macrophage (25). SPP1 mediates immune cell adhesion, migration, activation, anti-apoptosis, and other biological functions, suggesting that it is a potential mediator of VC (26). Thus, this review summarized the potential function of SPP1 in VC through the regulation of immune cells.

## SPP1 signaling and immune cells

### Immune cell infiltration

**Figure 1:**
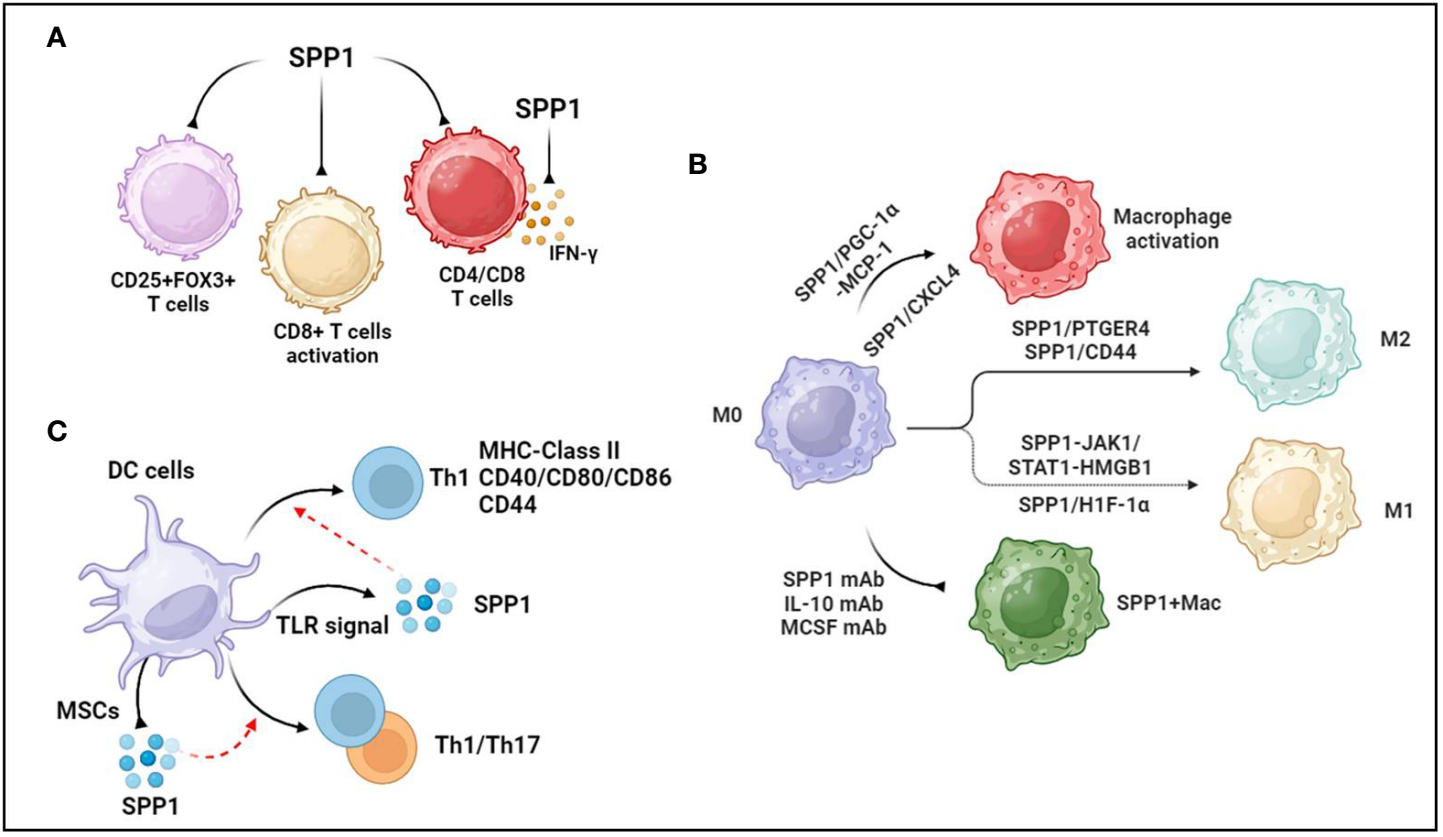
Association between SPP1 and immune cells. **(A)** SPP1 reduces regulatory T cells, inhibits CD8+T cells activation and suppresses CD4+ and CD8+ T-cell infiltration. Additionally, SPP1 inhibits IFN-γ secretion. **(B)** PGC-1α increases SPP1 secretion from monocytes, mediating macrophage activation and recruitment through MCP-1 expression. Platelet-derived CXC chemokine ligand 4 (CXCL4) is required for SPP1+ macrophage activation. Anti-SPP1, anti-IL-10, and anti-MCSF antibodies reduce the number of SPP1+ macrophages. During macrophage polarization, SPP1 induces the polarization of macrophages to M2-like TAMs through SPP1/CD44 and SPP1-PTGER4 signaling. SPP1 stimulates Janus kinase 1/signal transducers and activators of transcription 1 (JAK1/STAT1) signaling in hepatocytes to produce high-mobility group box 1 (HMGB1), which facilitates macrophage polarization toward the M1 phenotype. Upregulated SPP1 promotes M1 macrophage polarization through the overexpression of hypoxia-inducible factor 1α (HIF-1α). **(C)** TLR signaling promotes the production of SPP1 in dendritic cells. SPP1 induces DC differentiation toward the T helper 1 (Th1) phenotype, accompanied by increased MHC class II, costimulatory (CD40, CD80, and CD86), and adhesion molecule (CD44) levels. Activated mesenchymal stromal cells (MSCs) reduce the level of SPP1 generation by DCs cocultured with IL-1β, IL-6, and TNFα, but increased levels of SPP1 in CD103-DCs induce Th1 and Th17 immune cell responses during experimental colitis. The figures were generated with BioRender (https://biorender.com/).

SPP1 is regarded as a key mediator of cell adhesion and migration, and SPP1 binding to OPN receptors (integrins and CD44) promotes cell migration and effector functions (27). A recent study showed that CD4^+^ T cells generating SPP1 exerted a beneficial effect on controlling acute graft-versus-host disease (aGVHD) through limiting gastrointestinal pathology (a major target organ of aGVHD) in a mouse model of aGVHD (28). In addition, immune correlation analysis revealed that SPP1 expression was higher in resting CD4 memory T cells and lower in regulatory T cells in the diagnosis of biliary atresia, suggesting that SPP1 mediated CD4+ T-cell and regulatory T-cell infiltration (29) (**Figure 1A**). Additionally, integrated analysis of bulk and single-cell RNA sequencing data showed that SPP1 was positively associated with myeloid cell infiltration but negatively associated with CD4/CD8 cell infiltration in the prognosis of patients with hepatocellular carcinoma (30) (**Figure 1A**).

Similarly, SPP1 is positively correlated with CD8 + cells, CD4 + cells, macrophages, neutrophils, and DCs in ovarian cancer (31). In the context of intrahepatic cholangiocarcinoma (iCCA), there were more SPP1+ macrophages that infiltrated the peripheral small duct type of S100P-SPP1 + iCCA (32). A study in OPN/SPP1 knockout mice showed that there was disorganized wound remodeling and defective macrophage infiltration after injury or infection (33). Furthermore, OPN-deficient DCs transduced with SPP1 could rescue Th17 cell generation *in vivo* and *in vitro* (34). On the other hand, intracellular OPN (iOPN) decreases the population size of myeloid progenitor cells and myeloid cells, and secreted OPN (sOPN) increases the population size of lymphoid cells (35). Supernatants of CD153+PD-1+CD4+ T cells remarkably promoted macrophage migration in a cell migration assay, which was inhibited in the presence of an anti-SPP1 antibody (36). Interestingly, OPN treatment during *C. neoformans* infection sharply increased the number of pulmonary eosinophils but decreased the total number of neutrophils without affecting the number of CD4+ T cells, DCs, or alveolar macrophages (37). These data demonstrate that OPN/SPP1 not only regulates immune cell infiltration but also controls immune cell differentiation.

### SPP1 and T-cells

OPN inhibits the activity of cytotoxic CD8+T lymphocytes (CTLs), contributing to the progression of malignant disease (38). OPN is expressed in myeloid regulatory cells (MRCs) and malignant cells, which are two major components of the tumor microenvironment. SPP1 suppressed IFN-γ secretion by binding to CD44, which was more highly expressed in activated T lymphocytes (38) (**Figure 1A**). Another study showed that SPP1 decreased CD69+CD8+ T cells, CD25−CD8+ T cells, and PD-1+CD8+ T cells, suggesting that the SPP1 protein likely suppressed CD8+ T-cell activation (39) (**Figure 1A**). Knocking out SPP1 in colon tumor cells increased the CTL lytic activity of T cells *in vitro* and inhibited tumor growth *in vivo*, whereas the protein level of OPN increased in the peripheral blood of tumor-bearing mice (40). A recent study showed that macrophage-specific deletion of SPP1 strengthened the efficacy of anti-PD-1 treatment in liver cancer and reduced cancer-associated fibroblasts (CAFs) infiltration and increased cytotoxic T-cell infiltration (41). Additionally, SPP1 knockout expanded granulocyte-oriented myeloid-derived suppressor cells (MDSCs), which was associated with the inhibition of lung metastases (42). Further data suggested that SPP1 deletion decreased the amount of regulatory T-cell accumulation at the metastatic site.

### SPP1 and macrophages

Given that SPP1 is predominantly secreted from macrophages, SPP1+ macrophages are widely recognized to regulate various diseases, such as cancers (31), cardiovascular diseases (43), tissue fibrosis (44, 45), and nonalcoholic steatohepatitis (46). Another study demonstrated that PGC-1α increased SPP1 secretion from monocytes, mediating macrophage activation and recruitment through MCP-1 expression (47) (**Figure 1B**). Moreover, secreted SPP1 regulates monocyte/macrophage biology (48, 49). As a case in point, upregulated SPP1 promoted macrophage polarization toward the M1 phenotype but not the M2 phenotype through the overexpression of hypoxia-inducible factor 1α (HIF-1α) (50) (**Figure 1B**). In colorectal cancer, SPP1+ macrophages highly express complement component 1C chain (C1QC), mannose receptor C type 1 (MRC1), signal transducer and activator of transcription 1 (STAT1), and peroxisome proliferator-activated receptor gamma (PPARG), which are associated with macrophage polarization (51). Furthermore, single-cell RNA-seq analysis demonstrated that SPP1 controlled the interaction between HCC cells and macrophages through SPP1/CD44 and SPP1-PTGER4 signaling, and *in vitro* data also showed that SPP1 induced the polarization of macrophages to M2-like tumor-associated macrophages (TAMs) (52) (**Figure 1B**). However, SPP1 stimulated Janus kinase 1/signal transducers and activators of transcription 1 (JAK1/STAT1) signaling in hepatocytes to secrete high-mobility group box 1 (HMGB1), which facilitated macrophage polarization toward the M1 phenotype (53) (**Figure 1B**). Nevertheless, a recent study suggested that compared with monocytes and MARK4+ macrophages, macrophages expressing both SPP1^High^ and CXCL9^High^ TAMs exhibit upregulated M0, M1, and M2 phenotype markers in human cancers, but the expression levels of M2 markers were higher than those of M1 markers in SPP1^High^ TAMs (54). Thus, the roles of SPP1 in macrophage polarization should be further investigated.

In terms of tissue-resident macrophages, SPP1 is known as a key mediator of IL-10–STAT3–Galectin-3 axis signaling in cardiac macrophages after MI (15). Further study revealed a reduction in the number of SPP1-producing macrophages following the administration of the anti-IL-10 antibody alone or the coadministration of the anti-IL-10 antibody plus the anti-MCSF antibody after MI (55) (**Figure 1B**). Senescent fibro-adipogenic progenitors actively increased macrophage recruitment *in vitro*, which inhibited the recruitment of macrophages after anti-SPP1 antibody treatment (56) (**Figure 1B**). The data showed that SPP1 regulated macrophage recruitment during the senescence of fibro-adipogenic progenitors. Additionally, platelet-derived CXC chemokine ligand 4 (CXCL4) is required for SPP1+ macrophage activation and organ fibrosis, and an expansion in SPP1+ macrophages of patients with CKD and those with heart failure has also been found (57) (**Figure 1B**). IL-6 secreted from tumor enteric glial cells (EGCs) facilitates monocyte differentiation toward SPP1+TAMs (58). There was an increase in the expression level of SPP1 in silica-treated RAW264.7 macrophages (59). These data suggested that SPP1 expression was associated with cytokines and materials, thus potentially offering solutions to human diseases by regulating SPP1+ macrophages.

### SPP1 and dendritic cells

DCs, known as a source of SPP1, are central mediators of the induction of T-cell immunity. SPP1 regulates the migration, differentiation, maturation, and survival of DCs (60–62). In addition, TLR signaling modulated the production of SPP1 in DCs (63) (**Figure 1C**). There was a significant association between SPP1 and DC markers in ovarian cancer, where SPP1 regulated DC infiltration (31). The cytokine milieu mediates the production of SPP1 in DCs, including IL-27, IFN-I, IL-1β, IL-6, and TNFα (34, 64). SPP1 induced DC differentiation toward the T helper 1 (Th1) phenotype, accompanied by increased MHC class II, costimulatory (CD40, CD80, and CD86), and adhesion molecule (CD44) levels (60) (**Figure 1C**). There was an increase in the level of SPP1 in CD103-DCs during experimental colitis, which induced Th1 and Th17 immune cell responses (65) (**Figure 1C**). Activated mesenchymal stromal cells (MSCs) reduced the level of SPP1 generation by DCs cocultured with IL-1β, IL-6, and TNFα (64) (**Figure 1C**). These data demonstrated that SPP1 mediated the biological functions of DCs, which promoted DC-induced immune responses in various diseases.

## The association between SPP1 and immune cells in vascular calcification

### Cardiovascular calcification

**Figure 2:**
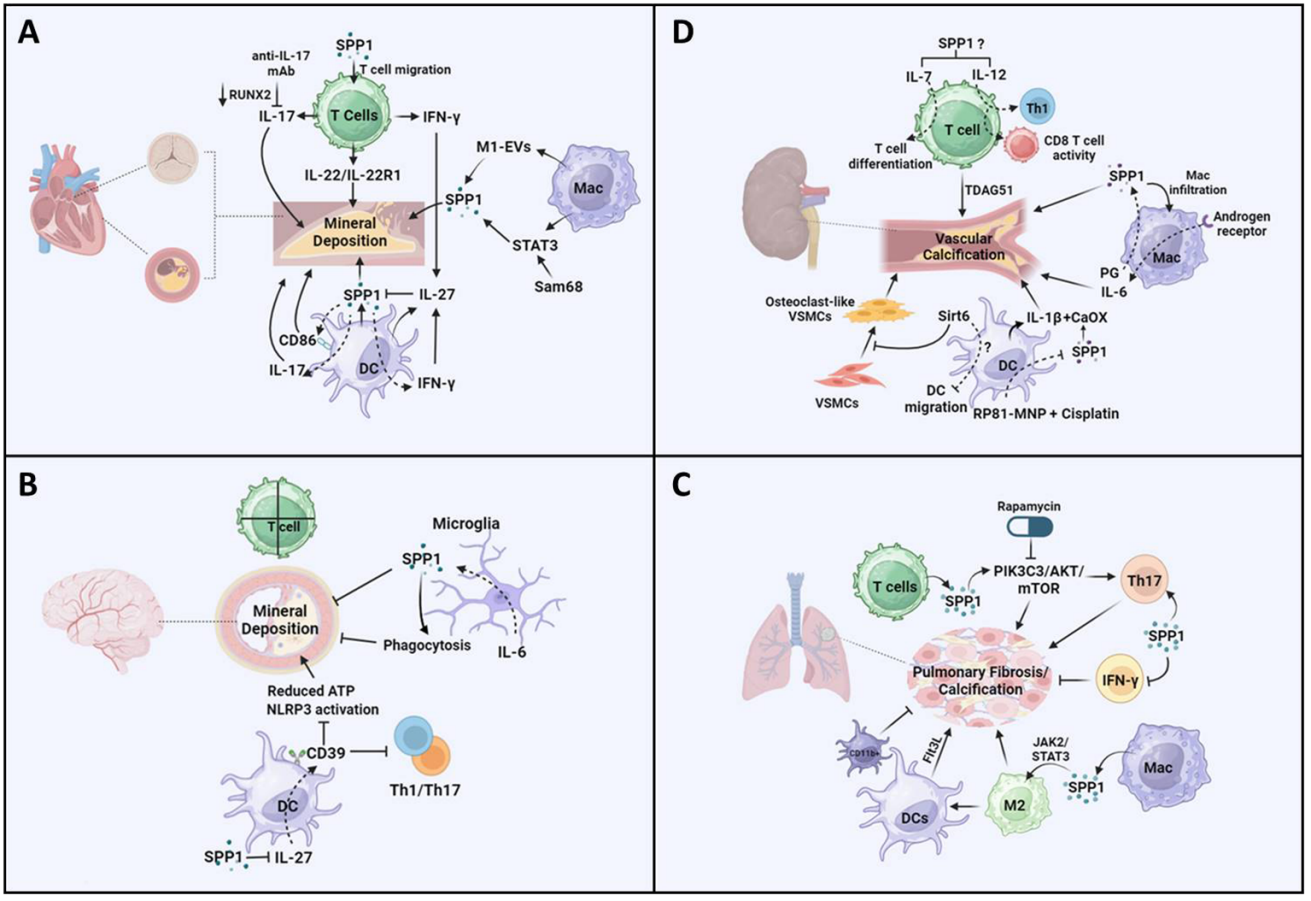
Potential pathways of SPP1-mediated T cells, macrophages, and dendritic cells in the progression of vascular calcification. **(A)** Increased production of SPP1 is detected in senescent CD153^+^PD-1^+^CD44^hi^CD4+ T cells in diabetic mice and consequently may induce IFN-γ/IL-17. SPP1 resulted in CD4+ and CD8+ T-cell infiltration, IL-22 promotes mineral deposition and osteoblastic differentiation via IL-22R1. There was a reduction in valve calcium deposition accompanied by reduced RUNX2 expression in response to IL-17A-neutralizing antibody treatment. Extracellular vesicles (EVs) derived from M1-polarized macrophages (M1-EVs) enhanced the expression of osteogenesis-related genes such as SPP1 and calcium nodule formation. Increased macrophage infiltration regulates STAT3 to promote osteogenic calcification. Src-associated in mitosis 68-KD (Sam68) coordinated with STAT3 in human CAV, resulting in mineral deposition via SPP1 signaling. CD86 expression has been found in DCs in coronary artery disease, and SPP1 expression is positively related to CD86. IFN-γ increased IL-27 expression and decreased SPP1 expression in DCs, and the inhibitory effect of IL-27 on SPP1 was detected. **(B)** There is no evidence showing that T cells induce vessel calcification in the brain. Upregulated SPP1 is detected in cerebellar microglia from the CNS-targeted production of IL-6 (GFAP-IL6 mice). Moreover, SPP1-producing microglia inhibit the progression of peripheral ectopic calcification *in vivo.* SPP1 suppressed the expression of IL-27 in DCs. IL-27 expression is detected in astrocytes in multiple sclerosis (MS) brains. IL-27 increases the expression of CD39 on DCs, which enhances tolerance by inhibiting Th1- and Th17-induced immune responses. There was a reduction in the concentration of ATP and activation of the NLRP3 inflammasome pathway. **(C)** Calcified fibrotic nodules that resorbed non-active lesions contained fewer CD3+ T cells, producing SPP1. SPP1 stimulates the PIK3C3-AKT-mTOR pathway, promoting chronic inflammation and Th17 differentiation. Rapamycin inhibits the progression of mesenchymal stromal cell calcification. Additionally, SPP1 increased the ratio of IL-17-producing T cells to IFN-γ-producing T cells, resulting in lung fibrosis. SPP1 regulates macrophage polarization toward the anti-inflammatory M2 phenotype via upregulation of the Janus kinase 2 (JAK2)/STAT3 signaling pathway, resulting in pulmonary fibrosis. Additionally, a higher expression level of SPP1 regulates macrophage polarization toward the M2 phenotype, resulting in the activation of dendritic cell infiltration in patients with lung adenocarcinoma with EGFR mutations. The expression level of FMS-like tyrosine kinase-3 ligand (Flt3L) and the number of lung DCs increased significantly during the progression of pulmonary fibrosis, but the accumulation of CD11b-positive cells inhibited lung fibrosis in a mouse model. **(D)** TDAG51 (T-cell death-associated gene 51) is a key predictor of vascular calcification in patients with chronic kidney disease. There is an increase in the expression of IL-7 and IL-12 in chronic kidney disease, which promotes T-cell differentiation and CD8+ cytotoxic T cells. In macrophages, there was a reduction in macrophage infiltration and kidney fibrosis in SPP1 KO mice following ischemia-reperfusion injury. Targeting the androgen receptor suppresses phosphate-induced vascular smooth muscle cell calcification by decreasing IL-6 expression. PG treatment inhibits the reduced H3K4me3 modification of SPP1. IL-1β-producing dendritic cells are related to kidney stone formation, which is associated with CaOx crystals inducing an inflammatory response. SPP1 has consistently been recognized as one of the inner cores of CaOx deposits. There was a reduction in SPP1 expression in all kidney cells treated with MNP-encapsulated RP81 (RP81-MNPs) after cisplatin activation. Furthermore, Sirt6 alleviated vascular calcification in CKD by inhibiting the osteogenic transdifferentiation of VSMCs and that Sirt6 inhibited the progression of experimental autoimmune encephalomyelitis through a reduction in DC migration. The figures were generated with BioRender (https://biorender.com/).

Valve interstitial cells undergo myofibrogenesis, osteogenic differentiation, calcification, and mineralization in CAVD, which contributes to cardiac outflow obstruction (66). In addition to common risks (aging, obesity, diabetes, hypertension, smoking, etc.), plasma lipids, inflammation, mineralization, and fibrosis contribute to CAVD (67). More interestingly, immune cells play a vital role in CAVD, and the interaction between SPP1 and these immune cells may mediate the progression of CAVD (68). There was an increase in the level of SPP1 in the heart with age, and SPP1 is known as one of the hub genes in CAVD (69). Additionally, highly expressed SPP1 was found in the cardiac valve of adult sheep and was associated with the progression of CAVD during aging (70). An increasing number of studies have demonstrated that different T-cell subsets, such as T helper cells, CTLs, regulatory T cells, memory effector T cells, and natural killer T cells, are present in the aortic valve during the development of CAVD (71, 72). Another study demonstrated that SPP1 expression in the calcific aortic valve resulted in CD4+ and CD8+ T-cell infiltration, which, in turn, accelerated CAVD (73) (**Figure 2A**). Cytokines such as IL-22 and chemokines (CXCL9) are derived from T cells and promote CAVD (74, 75). Interestingly, IL-22 promoted mineral deposition and osteoblastic differentiation via IL-22R1 during mineralization in human CAVD (74) (**Figure 2A**). Furthermore, T cells predominantly secreted OPN (28) (**Figure 2A**). In diabetic mice, increased SPP1 production was detected in senescent CD153^+^PD-1^+^CD44^hi^CD4+ T cells (76). SPP1 also induced IFN-γ and IL-17 expression in T cells but inhibited IL-10 expression in T cells and B cells (77) (**Figure 2A**). Consequently, there was a reduction in valve calcium deposition accompanied by reduced RUNX2 expression in response to IL-17A-neutralizing antibody treatment (78) (**Figure 2A**). Therefore, there was an association between T cells and SPP1 in mediating the progression of CAVD. Additionally, a recent study showed that there has been a significant increase in the expression levels of SPP1, HMOX1, and CD28 in the CAVD group (25). Further data suggested that lymphocyte counts were correlated with CAVD. However, there is little evidence to support the mechanisms by which SPP1 affects T cells in CAVD.

Macrophages mainly participate in the progression of cardiovascular diseases, including atherosclerosis (79), myocardial infarction (80), cardiac repair (81), and CAVD (82). A recent study showed that SPP1+ macrophages promoted atrial fibrillation and that the level of SPP1 in macrophages increased during atrial fibrillation (43). Furthermore, studies in CAVD have suggested that macrophages secrete SPP1, which is associated with CAVD progression (25, 83). Aortic valve interstitial cells (AVICs) internalized DiI-labeled extracellular vesicles (EVs) derived from M1-polarized macrophages (M1-EVs), which promoted the expression of osteogenesis-related genes such as SPP1 and calcium nodule formation (84) (**Figure 2A**). On the other hand, SPP1 mediated IL-10–STAT3–Galectin-3 axis signaling in cardiac macrophages after MI (15) (**Figure 2A**). A study in Notch1+/− aortic valve disease demonstrated that macrophage infiltration increased and macrophage phenotype shifted toward pro-inflammatory macrophage, resulting in decreased STAT3β, which inhibited the expression of STAT3α and RUNX2 (82). Consequently, changes in these genes led to osteogenic calcification (82). Additionally, upregulated Src-associated in mitosis 68-KD (Sam68) expression was found in human CAV, which coordinated with STAT3, resulting in mineral deposition and osteogenic differentiation (85) (**Figure 2A**). These results showed that SPP1-Sam68-STAT3 signaling could mediate macrophages in CAVD.

DCs are one of the main leukocyte populations in heart valve leukocytes (86). A recent integrated bioinformatics analysis revealed that CD86 was upregulated in the aortic valve stenosis (87). In fact, the expression of CD86 has been found in DCs in coronary artery disease (88) (**Figure 2A**). Additionally, SPP1 expression is positively related to CD86, which is associated with M2-type macrophages in colorectal cancer (41). These data suggested that SPP1-CD86 signaling could modulate DC function to affect the progression of CAVD. Furthermore, SPP1 increased IFN-γ expression and the Th17/Treg ratio in different models (50, 89), but IFN-γ and IL-17 mediated DC migration and T-cell activation through DCs (90, 91). In turn, IFN-γ alleviated IL-17-induced autoimmune inflammation by increasing IL-27 expression and decreasing SPP1 expression in DCs, and a further study indicated that IL-27 inhibited the expression of SPP1 in DCs (92) (**Figure 2A**). Another study discovered that IL-27R knockout contributed to the accumulation of myeloid cells and T cells, resulting in severe atherosclerosis in mice (93). However, there are few concerns in CAVD through the differential regulation of SPP1 in DCs.

### Brain vascular calcification

Brain VC is largely related to aging and neurodegenerative and neuroinflammatory diseases (94). Recent studies have shown that stroke and myocardial infarction result in intracranial arterial calcifications, which are not associated with cognitive outcomes (95). Additionally, large artery stiffness causes brain vascular dysfunction, which is linked to an inflammatory response and increased oxidative stress (96). Accumulating studies have demonstrated that the expression level of SPP1 increases in various CNS disease models, which exerted an association between SPP1 and immune cells (27). Another study discovered that platelet-derived growth factor BB (PDGF-BB) phosphorylates its receptors PDGFRβ and ERK, resulting in RUNX2 activation (94). Additionally, SPP1 expression increased significantly in a platelet-derived growth factor BB (PDGF-BB)-induced brain VC model (94). SPP1 is expressed in the brain vasculature, while SPP1 is expressed in pericytes and fibroblast-like cells in the brain (97). In the context of immune cells, CD3+ T cells have been found in the surroundings of calcifications in the brain parenchyma, but there is no evidence showing that T cells induce vessel calcification in the brain (98) (**Figure 2B**). However, the expression of SPP1 and integrin subunit alpha X (ITGAX) is associated with cognitive decline and neuropathologies through the regulation of microglial subsets (99). Thus, further studies should investigate whether SPP1 expression during VC affects T-cell function or recruitment to promote mineral deposition during brain VC.

SPP1-producing macrophages play an important role in calcification. Monocytes differentiate into SPP1-producing macrophages via calcium, which contributes to the pro-inflammatory macrophage response (100). VCs are positively correlated with Alzheimer’s disease in elderly individuals (101). Additionally, a study in Alzheimer’s disease models suggested that SPP1 expression was required for microglial synaptic phagocytosis (102, 103) (**Figure 2B**). Thus, SPP1+ macrophages could modulate brain VC, thus representing a potential target for treating brain diseases in individuals of different ages. On the other hand, microglia limited calcification induced by IL-6- and IFN-α-mediated neuroinflammation, and upregulated SPP1 was detected in cerebellar microglia from CNS-targeted production of IL-6 (GFAP-IL6 mice) (104) (**Figure 2B**). Moreover, SPP1, which is produced in microglia, inhibited the progression of peripheral ectopic calcification *in vivo* (105, 106) (**Figure 2B**). Therefore, SPP1 may restrict calcium deposition through microglia, which could connect with the IL-6 signaling pathway.

Additionally, recent data have shown that different types of DCs have been found in cerebral ischemia, such as conventional type 1 DCs (cDC1s), conventional type 2 DCs (cDC2s), monocyte-derived DCs, migratory DCs, and plasmacytoid (103). Additional data also showed that there was an increased expression of SPP1 in four clusters of DCs after experimental stroke (103). Additionally, a previous study illustrated that SPP1 (iOPN) suppressed the expression of IL-27 in DCs but promoted that of Th17 cells (107) (**Figure 2B**). The expression level of IL-27 was detected in astrocytes in multiple sclerosis (MS) brains (108). Furthermore, IL-27 increased the expression of CD39 on DCs, which enhanced tolerance by inhibiting Th1- and Th17-induced immune responses (109) (**Figure 2B**). Further data showed that there was a reduction in the concentration of ATP and activation of the NLRP3 inflammasome pathway (109–111) (**Figure 2B**). These data demonstrated that secretion of SPP1 from DCs could regulate the progression of brain VC through IL-27 and CD39. Thus, more mechanisms by which SPP1 regulates DCs should be investigated in the context of brain VCs.

### Pulmonary vascular calcification

Pulmonary arterial hypertension (PAH) contributes to pulmonary VC, which is associated with impaired vascular stiffness, pulmonary artery atherosclerosis, and inflammation (4, 112–114). In the pulmonary artery, there was a reduction in SPP1 expression with age (70). A recent meta-analysis study showed that there was a higher increase in the expression level of SPP1 in patients with idiopathic pulmonary fibrosis (IPF) (70). Moreover, small calcified lung nodules frequently contribute to dystrophic calcification in injured lungs (115). Calcified fibrotic nodules that resorbed non-active lesions contained fewer CD3+ T cells (116). A great deal of CD3+ and CD4+ T cells have been found in other sites, such as closed necrotic, non-necrotic cellular granulomas, and central cavities of open granulomas (116). However, in terms of the cavity surface, there were no T cells in the necrotic zone or on the cavity surface, which contributed to preventing the interaction between macrophages and T cells (116). Furthermore, the role of SPP1 in T-cell migration, adhesion, and activation has been well investigated. Thus, SPP1 could regulate T cells to control the progression of calcified fibrotic nodules. Interestingly, an increase in the expression of SPP1 was found in acid fast bacilli (AFB)-scarce lesions (116). Additionally, increasing levels of IL-15 and SPP1 contribute to decreasing the number of bacteria in AFB-scarce lesions (116). These data showed that SPP1 plays an anti-microbial role in lung diseases (116). Another study demonstrated that inhibition of SPP1 blocked the PIK3C3-AKT-mTOR pathway, resulting in the alleviation of chronic inflammation and mediating Th17/Treg differentiation in chronic obstructive pulmonary disease (COPD) (117) (**Figure 2C**). Furthermore, rapamycin, an mTOR inhibitor, suppressed the progression of MSC calcification (**Figure 2C**) (118). Additionally, SPP1 increased the ratio of IL-17-producing T cells to IFN-γ-producing T cells, resulting in lung fibrosis (119, 120) (**Figure 2C**). These data suggest that T cells modulate lung inflammation and calcification via SPP1 signaling pathways.

Macrophages also affected lung calcifications, which are divided into two types of tissue-resident macrophages [alveolar macrophages (AMs) or interstitial macrophages (IMs)] (121). There was an increase in the number of SPP1^high^ macrophages in fibrotic lungs rather than FABP4^high^ and FCN1^high^ (122). Another study also showed that SPP1-expressing monocytes/macrophages were mainly found around microliths in patients with pulmonary alveolar microlithiasis (PAM) (123). Interestingly, SPP1 regulates macrophage polarization toward the anti-inflammatory M2 phenotype via upregulation of the Janus kinase 2 (JAK2)/STAT3 signaling pathway, resulting in pulmonary fibrosis (**Figure 2C**) (124). On the other hand, the expression level of MERTK is highly increased in SPP1^high^ macrophages, which could be a potential treatment for IPF (125). MERTK was highly elevated in macrophages, promoting profibrotic effects in pulmonary fibrosis (126). These data showed that MERTK^high^ SPP1^high^ macrophages could regulate pulmonary fibrosis and consequently result in lung calcification.

Additionally, increased SPP1 expression regulated macrophage polarization toward the M2 phenotype, which resulted in the activation of DC infiltration in patients with lung adenocarcinoma with EGFR mutations (127) (**Figure 2C**). Additionally, the expression level of FMS-like tyrosine kinase-3 ligand (Flt3L) and the number of lung DCs increased significantly during the progression of pulmonary fibrosis in both mice and humans, but the accumulation of CD11b-positive cells inhibited lung fibrosis in a mouse model (**Figure 2C**) (128). Another study suggested that there was a direct association between SPP1 and KRAS mutation in lung cancer and that SPP1 deficiency had a protective effect on patients with KRAS mutation in lung cancer (129). An increased number of DC1s and eosinophils was detected in the advanced-IPF group compared with the control group or the early-IPF group, demonstrating that these immune cells may modulate the late stage of IPF (130). However, the effects of SPP1-mediated DCs on lung calcification have been less studied.

### Chronic kidney diseases

VC, which injures endothelial cells and vascular smooth muscle, is an important factor that induces morbidity and mortality in patients with CKD (131). Compared with those in the normal group, calcium salts are prone to pathological deposition in the arterial wall of patients with CKD (132). Additionally, elevated extracellular phosphate, severe inflammation, and cellular senescence lead to VC in CKD patients (132). In the context of the inflammatory response, a recent study demonstrated that TDAG51 (T-cell death-associated gene 51) was a key predictor of VC in patients with CKD (133) (**Figure 2D**). IL-7 is a key cytokine involved in T- and B-cell development and regulates the immune response in various diseases (134) (**Figure 2D**). Additionally, there was an increase in the expression level of IL-7 in CKD (**Figure 2D**). The level of IL-12, another cytokine, was also increased in CKD, which differentiated naive T cells into Th1 subsets and promotes CD8+ cytotoxic T-cell and NK cell activity (135, 136) (**Figure 2D**). However, no association between SPP1 and VC via the IL-7 or IL-12 signaling pathway has been reported in CKD. In addition, microglia treated with SPP1 exhibited a decrease in IL-6 expression. sIL-6 is associated with VC and chronic inflammation in CKD (137). Thus, the effects of SPP1/IL-6 on T cells to regulate kidney VC should be investigated.

Furthermore, there was a reduction in macrophage infiltration and kidney fibrosis in SPP1 KO mice following ischemia-reperfusion injury (138) (**Figure 2D**). Targeting macrophage androgen receptor decreased IL-6 expression, which suppressed phosphate-induced VSMC calcification (139) (**Figure 2D**). Epigenetic regulation is involved in inflammation and CAD progression, and histone methyltransferase inhibitors alleviate the progression of the pro-inflammatory response by switching macrophages to foam cells (140, 141). Additionally, a study of E3 ligase von Hippel–Lindau protein (VHL)-deficient macrophages showed that 3-phosphoglyceric acid (PG) treatment inhibited the reduced H3K4me3 modification of SPP1, resulting in increased SPP1 expression (142, 143) (**Figure 2D**). Thus, epigenetic regulation of SPP1+ macrophages could be an effective target in VC. In the context of kidney stone disease (KSD), SPP1 was extensively associated with other hub genes among the 30 hub genes, which may be an interaction with macrophages to regulate KSD (144). Additionally, a study in OPN knockout mice with glyxylate administration suggested that OPN contributed to the formation of kidney stones, which was also associated with macrophage activity (145). During mineral deposition, SPP1 expression was detected in surrounding mineralized tissues in patients with KSD. More interestingly, large numbers of M1 and M1/M2 macrophages as well as T cells were found in the same area. However, this study did not confirm the correlation between SPP1 and immune cells related to mineral deposition or whether SPP1 recruited immune cells to plaques or SPP1+-producing macrophages promoted mineral deposition during the development of fibrosis (146). Another study demonstrated that calcium promoted monocyte differentiation into SPP1+ macrophages (100). Thus, targeting SPP1+ macrophages could be a potential therapy for the formation of kidney VC, and other regulatory mechanisms of SPP1+ macrophages should be studied in the future.

A recent study also revealed four subclusters of DC populations among kidney immune cells (147). IL-1β-producing DCs are also related to kidney stone formation, which is associated with CaOx crystals inducing an inflammatory response (148–151) (**Figure 2D**). Furthermore, SPP1 has consistently been recognized as one of the inner cores of CaOx deposits (152) (**Figure 2D**). In terms of cisplatin-induced CKD, the inflammatory response was enhanced in the RNLS-KO model, especially for those DC populations increased to fivefold compared with that in the wild type (153). Moreover, further data showed that there was a reduction in SPP1 expression in all kidney cells treated with MNP-encapsulated RP81 (RP81-MNPs) after cisplatin activation (153) (**Figure 2D**). However, there was no evidence that SPP1 was associated with DCs in this kind of model. Sirt6 inhibited the progression of experimental autoimmune encephalomyelitis through a reduction in DC migration (154) (**Figure 2D**). Furthermore, a study in SIRT6-transgenic (SIRT6-Tg) mice demonstrated that Sirt6 alleviated VC in CKD by inhibiting the osteogenic transdifferentiation of VSMCs (155) (**Figure 2D**). Therefore, the association between SPP1 and other signaling pathways (IL-1β and Sirt6) in DCs should be addressed more specifically in the future, which could provide an effective target for VCs.

## Conclusions and future perspectives

Vascular diseases are the main cause of mortality in humans, and accumulating studies have confirmed that VC is involved in the inflammatory response in human diseases and that SPP1-mediated immune cells play an important role in the progression of VC. The specific mechanisms by which SPP1 mediates immune cells and VC should be adequately addressed, as SPP1 could be a potential therapeutic target for VC.

In recent decades, the role of the inflammatory response in VC has been well reported. However, the regulatory effects of SPP1 on immune cells in VC remain unclear. In this review, we summarize the effects of SPP1 on T cells, macrophages, and DCs to decipher the association between SPP1 and these immune cells in VC. Identifying specific regulatory mechanisms between SPP1 and immune cells is essential because these mechanisms contribute to VC in different diseases. The SPP1–cytokine–T-cell axis might be an important mediator in the progression of VC in different organs. Cytokine-neutralizing antibodies, T-cell antibodies, or mTOR inhibitors could offer potential therapeutic strategies to alleviate VC by regulating mineral deposition in patients. Moreover, SPP1+ macrophages have been identified as significant immune cells in VC. SPP1/IL-6 signaling can regulate macrophages to affect VC progression, and targeting SPP1/IL-6 signaling provides a unique method to decipher the different mechanisms involved in the formation of VC. Additionally, targeting SPP1/STAT3 pathway signaling in macrophages plays a vital role in VC development, which could mediate the progression of mineral deposition and osteogenic differentiation. On the other hand, the effects of SPP1 on DCs should be further investigated in the context of VC, given that interactions between SPP1 and DCs have been detected in diseased tissues.

## Author contributions

YZ: Conceptualization, Formal analysis, Funding acquisition, Investigation, Methodology, Project administration, Resources, Supervision, Visualization, Writing – original draft, Writing – review & editing. ZH: Conceptualization, Data curation, Formal analysis, Methodology, Validation, Visualization, Writing – original draft. LG: Conceptualization, Data curation, Formal analysis, Methodology, Validation, Visualization, Writing – original draft. HM: Formal analysis, Validation, Visualization, Writing – original draft. RC: Project administration, Resources, Supervision, Writing – original draft.
